# Case Report: Hyperinflammatory Status in an Immunocompromised Child With a Highly Sustained Viral Load of SARS-CoV-2

**DOI:** 10.3389/fmed.2021.675282

**Published:** 2021-08-19

**Authors:** Matias Moragas, Sandra Gomez, María Florencia Fernández, Marcelo Dario Golemba, Marcela Palladino, Daniela Borgnia, Silvina Ruvinsky, Lidia Fraquelli, Ana Buchovsky, Rosa Bologna, Andrea Mangano

**Affiliations:** ^1^Unidad de Virología y Epidemiología Molecular - CONICET, Hospital de Pediatría “Prof. Dr. Juan P. Garrahan”, Ciudad Autónoma de Buenos Aires, Argentina; ^2^Servicio de Epidemiología e Infectología, Hospital de Pediatría “Prof. Dr. Juan P. Garrahan”, Ciudad Autónoma de Buenos Aires, Argentina; ^3^Unidad de Cuidados Intermedios y Moderados, Hospital de Pediatría “Prof. Dr. Juan P. Garrahan”, Ciudad Autónoma de Buenos Aires, Argentina; ^4^Centro de Atención Integral del Paciente Hemato-Oncológico (CAIPHO), Hospital de Pediatría “Prof. Dr. Juan P. Garrahan”, Ciudad Autónoma de Buenos Aires, Argentina; ^5^Laboratorio de Serología, Hospital de Pediatría “Prof. Dr. Juan P. Garrahan”, Ciudad Autónoma de Buenos Aires, Argentina

**Keywords:** SARS-CoV-2, COVID-19, viral load, immunocompromised state, hyperinflammatory status, pediatrics

## Abstract

Coronavirus disease 2019 (COVID-19) is spreading throughout the world. Limited data are available for the dynamics of severe acute respiratory syndrome coronavirus 2 (SARS-CoV-2) viral load (VL) in immunocompromised pediatric patients. Here, we report the clinical characteristics and the dynamics of SARS-CoV-2 VL of a pediatric patient with acute myeloid leukemia who developed a hyperinflammatory status mimicked MIS-C. The clinical course was characterized by the late onset of fever, GI symptoms, rash, and respiratory distress, including oxygen requirement with sustained VL of SARS-CoV-2 around 7 log_10_ RNA copies/mL for 6 weeks. It is important to note that the hyperinflammatory status developed early at the third week of hospitalization—in a context of high VL and immunocompromised status. All these characteristics make this clinical case unique. On the other hand, while many reports have characterized the dynamics of SARS-CoV-2 VL in adults and immunocompetent hosts, it remains unreported in pediatrics—even less in immunosuppressed children.

## Introduction

In April 2020, serious cases of children with systemic hyperinflammatory status with a temporary association with SARS-CoV-2 infection were reported in the United Kingdom (UK) (1). Since then, pediatric cases with a similar condition have been reported in Europe, South Africa, and America (2–4). This entity identified as multisystem inflammatory syndrome in children (MIS-C) has a spectrum of manifestations similar to Kawasaki disease, toxic shock syndrome, sepsis, and macrophage activation syndrome. The World Health Organization (WHO), the Center for Disease Prevention and Control (CDC), and the Royal College of Pediatrics and Child Health (RCPCH) in the UK issued definitions for case identification (5–7). Although MIS-Cs were reported in immunocompetent patients, so far there are no records in the literature about this condition in immunosuppressed children.

Here, we report a case of an immunosuppressed child who developed a hyperinflammatory status temporally associated with SARS-CoV-2 infection in the presence of sustained high viral load (VL).

## Case

A 3-year-old male patient diagnosed with M7 acute myeloid leukemia in January 2020 in Paraguay was admitted to the Hospital de Pediatria Garrahan (Buenos Aires, Argentina) in February 2020 for treatment and follow-up. By the end of July, the child had completed the first and second intensification block of chemotherapy with cytarabine and etoposide to keep the patient in complete remission, awaiting a bone marrow transplant. On August 4th, due to a close contact with a person with COVID-19, the child and his mother were tested. A reverse-transcriptase–polymerase-chain-reaction (RT-PCR) assay for SARS-CoV-2 in nasopharyngeal swab specimen was performed with detectable results in both cases. The child was asymptomatic for COVID-19, but he was immediately hospitalized for isolation following national health policies at that time.

The child remained asymptomatic for 28 days, when beginning with febrile neutropenia. Peripheral blood and urine cultures were negative for bacterial and fungal infection. Galactomannan antigen tests in serum were negative. Other respiratory viruses including influenza A and B, adenovirus, parainfluenza 1, 2, 3, and 4, respiratory syncytial virus, metapneumovirus, rhinovirus, enterovirus, and pan-coronavirus (NL63, 229E, OC43, and HKU1) were also negative by molecular tests. He started broad-spectrum antibiotic treatment with piperacillin tazobactam associated with amikacin. On day 31, the child started coughing and had dysphonia. Twenty-four hours later, he started getting abdominal pain, diarrhea, and a maculopapular rash affecting the face and high upper thoracic area without compromised hand and feet. Laboratory tests showed that pancytopenia (white blood cells 100/mm^3^, hemoglobin 6.2 g/dl, platelets 8,000/mm^3^), fibrinogen 526 mg/dl, ferritin 4489.3 ng/ml, CPK (creatine phosphokinase) 14 UI/l, NT-PROBNP (N-terminal portion of pro-B-type natriuretic peptide) 226 pg/ml, C-reactive protein 136.14 mg/l, procalcitonin 1.22 ng/ml, normal hepatogram, and anti SARS-CoV-2 antibodies were negative. Detailed laboratory markers are shown in Table 1. No evidence of coagulopathy was shown. Myocardial dysfunction, pericarditis, valvulitis, or coronary abnormalities were discarded by echocardiography. In the presence of high inflammatory laboratory markers, acute SARS-CoV-2 severe infection or MIS-C was suspected. Gamma globulin treatment with a dose of 2 g/kg was immediately administered.

**Table 1:** Laboratory characteristics along SARS-CoV-2 infection.

	**Days post-infection**								
**Laboratory markers**	**28**	**31**	**32**	**33**	**34**	**35**	**38**	**49**	**64**
Absolute neutrophil count (×10^9^/l)	0	0	0	0	0	1,107	750	4,515	1,496
Absolute lymphocyte count (×10^9^/l)	0	0	0	0	0	305	64	531	528
Platelet count (×10^9^/l)	47,000	8,000	37,000	18,000	29,000	27,000	23,000	112,000	136,000
C-reactive protein (mg/l)	-	136.14	152.09	181.51	250.05	223.45	77.52	1.38	2.36
VSG (mm/h)	-	104	93	85	110	115	78	10	25
Ferritin (ng/ml)	-	4,489.30	-	-	16,955.38	23,899.44	16,394.78	5,351.16	3430.68
Procalcitonin (ng/ml)	-	0.63	0.77	-	1.22	0.60	0.16	-	-
PT (%)/PTT (s)	-	-	68/35	81/38	79/46	78/49	74/49	97/25	94/33
Fibrinogen (mg/dl)	-	526	571	513	530	486	447	158	274
Troponin (ng/ml)	-	5.0	5.0	-	6.8	6.8	3.5	-	8.6
ProBNP (pg/ml)	-	226	1,893	1,098	835	512	1,088	135	8.4
Urea (mg/dl)	-	13	18	17	10	14	16	39	20
Creatinine (mg/dl)	-	0.37	0.37	0.34	0.36	0.34	0.30	0.33	0.34
AST/ALT (IU/ml)	-	13/10	18/8	19/9	29/13	43/25	26/22	31/74	22/27
Albumin (g/dl)	-	3.64	-	-	2.70	3.10	3.16	4.10	-
Triglycerides (mg/dl)	-	54	67	-	105	99	-	88	61
CPK (IU/l)	-	-	19	-	14	13	-	-	-

On day 33, febrile neutropenia persisted, and the child started bronchospasms and hypoxemia requiring oxygen therapy by nasal cannula. Blood cultures were performed, and antibiotics were switched to meropenem, vancomycin, and lipid formulation of amphotericin B. A chest CT scan showed (i) patched areas of airspace occupation with confluent sectors and ground glass opacity in both upper lobes and (ii) extensive areas of consolidation with bronchogram in both lower lobes. A second echocardiogram showed no abnormal findings. On day 34, the patient remained feverish with diarrhea and abdominal pain and started with hypoxemia and vomits.

At day 35, the patient had a normal neutrophil count but remained lymphopenic. Due to worsening rash and respiratory symptoms, the child required a non-rebreathing mask and was admitted to the pediatric intensive unit (PICU). During the PICU stay, the patient did not require inotropic drugs or mechanical respiratory support. A 10-day treatment with dexamethasone 0.6 mg/kg per day was started, and convalescent plasma at a dose of 5 ml/kg was administered with good tolerance. The administration of convalescent plasma was in the context of institutional approved protocol. On day 38, the child was without fever and decreased oxygen requirements, going from PICU to an intermediate care unit. At day 53, the child was discharged with normalized laboratory markers, except for lymphopenia and high ferritin (Table 1).

**Figure 1:**
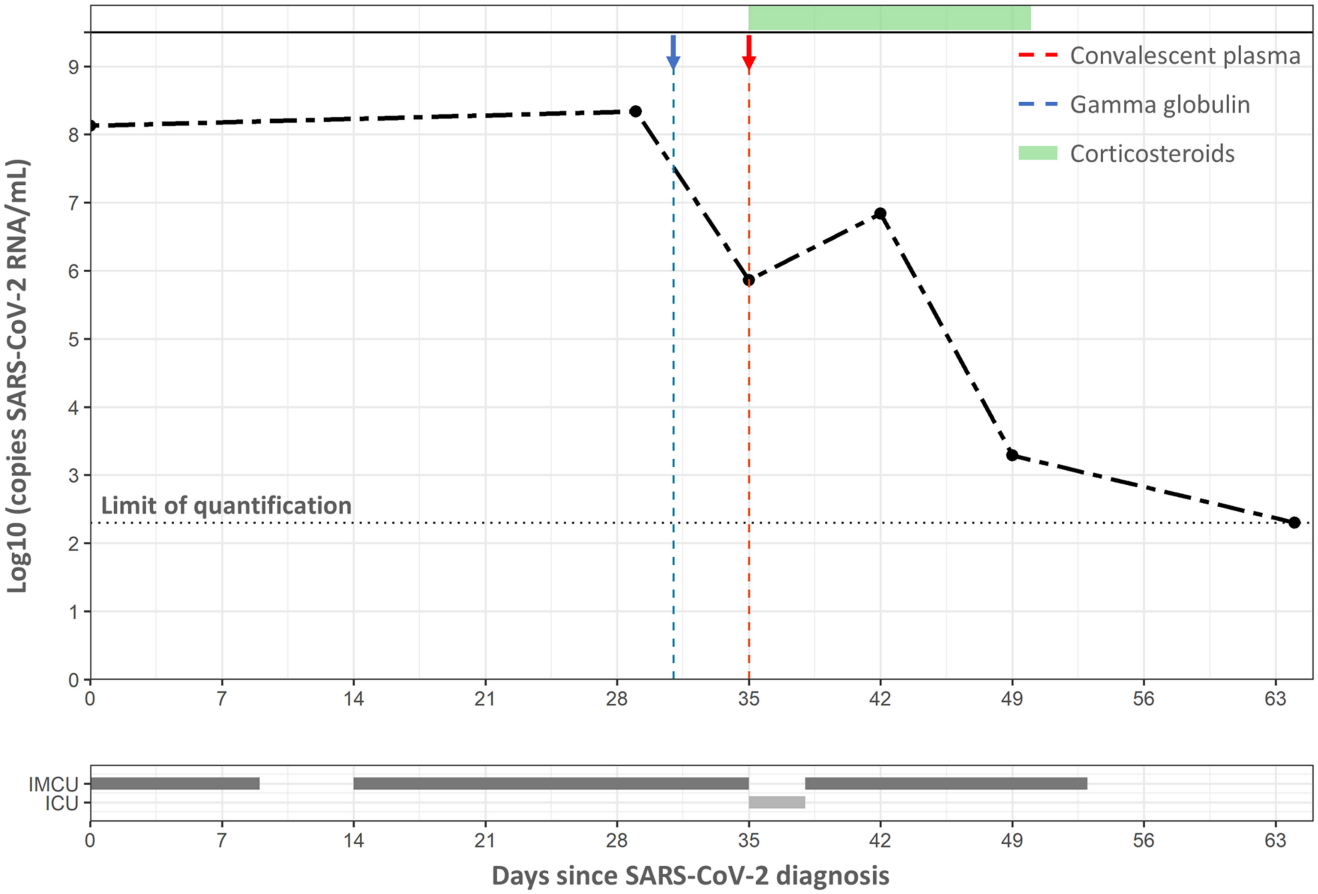
Dynamic of SARS-CoV-2 since admission. Dashed lines in gray, blue, and red represent the limit of quantification value, gamma globulin treatment, and convalescent plasma treatment, respectively, whereas the green bar represents the days under corticosteroids therapy. The bars below the VL curve show the period in which the child stayed in the intermediate care unit (IMCU) and intensive care unit (ICU).

SARS-CoV-2 VL monitoring was performed in nasopharyngeal swabs using an *in-house* quantitative RT-PCR, which was validated according to the guidelines proposed by Burd et al. (8) and Bustin et al. (9). It is important to mention that our assay included the measure of a housekeeping gene (RNase P) cycle threshold (Ct) to correct the specific-SARS-CoV-2 Ct—before its extrapolation from the standard curve—according to the number of cells in the samples and as recommended by Han et al. (10). The dynamic of SARS-CoV-2 VL was reconstructed in six samples along the 64 days of hospitalization (Figure 1). In summary, the child had a VL of 8.34 log_10_ copies per milliliter on the first day of symptom onset and remained above 8 log_10_ copies per milliliter and close to 7 log_10_ copies per milliliter until days 29 and 42 from admission, respectively. Then, VL decreased, reaching a value of 3.29 log_10_ copies per milliliter at day 49, and remained detectable but below the limit of quantification at day 64.

During the whole hospitalization, the patient did not show signs of hemodynamic instability or shock. After 3 months from hospital discharge, the patient received unrelated hematopoietic stem cells transplantation with normal clinical and laboratory markers. At present (June 2021), 6 months after transplantation, the child was in remission of his underlying disease with good clinical evolution.

The study was approved by the Institutional Review Board, and written informed consent was obtained from the parents.

## Discussion

Recently, a case report showed persistent infection of SARS-CoV-2 in an immunocompromised adult with confirmed infectious virus in nasopharyngeal samples from days 75 and 143 (11). Our case adds new evidence about persistently high VL of SARS-CoV-2 in an immunocompromised child, with diagnosis of acute severe infection of SARS-CoV-2 with hyperinflammatory status mimicking MIS-C. In acute severe infection and MIS-C, common features are gastrointestinal manifestations (abdominal pain, diarrhea), cutaneous signs (rash), inflammatory markers, and lymphopenia. However, children with severe COVID-19 mimicking MIS-C present more frequently with respiratory symptoms (e.g., cough, respiratory distress) while gastrointestinal symptoms are less common. Also, comorbidities (e.g., malignancy, chronic lung diseases, neurological disorders) are frequently associated with severe forms of disease as in the case reporting here (12). On the other hand, the clinical presentation of the inflammatory status in this patient appeared at the same time that MIS-C were usually diagnosed (3rd/4th week since SARS-CoV-2 diagnosis) (5–7). The majority of MIS cases—in children and adults—were diagnosed with negative SARS-CoV-2 RT-PCR, and detectable antibodies. It is important to highlight that in our case, the specific SARS-CoV-2 antibodies were negative, but it is an immunocompromised host (13). Up to our knowledge, this is the first immunocompromised child, with persistent high VL who developed a hyperinflammatory status mimicked MIS-C. Further studies are needed to unravel the host–virus interaction that led to hyperinflammatory status in an immunocompromised host.

## Data Availability Statement

There are no restrictions to apply to the data generated in this article. Requests to access the datasets should be directed to andreammangano@gmail.com.

## Ethics Statement

The studies involving human participants were reviewed and approved by Comité Revisor y de Ética en la Investigación, Hospital de Pediatría Garrahan. Written informed consent to participate in this study was provided by the participants' legal guardian/next of kin.

## Author Contributions

AM, DB, and MM conceived the study. SG, MP, DB, SR, LF, AB, and RB collected and analyzed the clinical data. MM, MF, and MG developed and performed the VL of SARS-CoV-2. MM, MF, MG, DB, and AM drafted the manuscript. All authors contributed and approved the final manuscript.

## Conflict of Interest

The authors declare that the research was conducted in the absence of any commercial or financial relationships that could be construed as a potential conflict of interest.

## Publisher's Note

All claims expressed in this article are solely those of the authors and do not necessarily represent those of their affiliated organizations, or those of the publisher, the editors and the reviewers. Any product that may be evaluated in this article, or claim that may be made by its manufacturer, is not guaranteed or endorsed by the publisher.
